# Complete chloroplast genome sequence of *Prunus mahaleb*

**DOI:** 10.1080/23802359.2019.1624217

**Published:** 2019-07-11

**Authors:** Xun Chen, Xin Shen, Dongyue Jiang

**Affiliations:** a College of Landscape Architecture, Northeast Forestry University, Harbin, China;; b Zhejiang Academy of Forestry, Hangzhou, China

**Keywords:** *Prunus mahaleb*, chloroplast genome sequence, phylogeny

## Abstract

*Prunus mahaleb* (L.) Mill. is a kind of ornamental species which originated from Europe and Western Asia. Here, we use Illumina paired-end sequencing to obtain the complete chloroplast genome of *P. mahaleb*. The complete cp genome of *P. mahaleb* with a size of 157,824 bp in length is a circular molecular genome, including a pair of inverted repeat (IR) region of 26,410 bp each, separated by a large single copy (LSC) region of 85,926 bp and a small single-copy (SSC) region of 19,078 bp. The genome consisted of 129 genes, including 84 protein-codon genes (PCGs), 37 tRNA genes, and eight rRNA genes. The GC content of the whole chloroplast genome is 36.7%. Phylogenetic analysis revealed that *P. mahaleb* was the closest related to *P. cerasoides* and *P. maximowiczii*.

## Introduction

*Prunus mahaleb* (L.) Mill. is originates from Europe and Western Asia, also cultivated in Hebei, Liaoning. It has been widely cultivated as an ornamental and commonly used as grafting stock for cherry. *P. mahaleb* thrive and tend to dwarf, also has the characteristics of drought and cold resistance. However, its chloroplast genome has not been reported. In this study, we first reported the complete chloroplast (cp) genome sequence of *P. mahaleb*.

Sample of *P. mahaleb* was collected from Tbilisi (Georgia; 44°49′40.78″E, 41°41′52.47″N). The specimen voucher (PE01682052) deposited in the Herbarium, Institute of Botany, Chinese Academy of Sciences. The total genomic DNA was extracted from the dried leaves using the mCTAB (modified cetyltrimethyl ammonium bromide) approach (Li et al. 2013). Paired-end reads of 2 × 150 bp were obtained with Illumina Hiseq PE150 Platform (Illumina, San Diego, CA). The chloroplast genome data were assembled *de novo* with SPAdes and extracted by using *P. cerasoides* (MF621234) as a reference, reconfirmed with Geneious (Bankevich et al. 2012; Kearse et al. 2012). and Plann and Sequin were used for initial annotation and correction (Huang and Cronk 2015).

After initial annotation, the putative starts, stops, and intron positions were determined by comparison with homologous genes in *P. cerasoides*. The complete cp genome of *P. mahaleb* with a size of 157,824 bp in length is a circular molecular genome, including a pair of inverted repeat (IR) region of 26,410 bp each, separated by a large single copy (LSC) region of 85,926 bp and a small single-copy (SSC) region of 19,078 bp. The genome consisted of 129 genes, including 84 protein-codon genes (PCGs), 37 tRNA genes, and eight rRNA genes. Among them, 17 genes occur in double copies and most of the gene occurs as a single copy. The whole chloroplast genome GC content is 36.7%, while the value of the LSC, SSC, and IR regions are 34.6%, 30.2%, and 42.5%, respectively.

**Figure 1.**
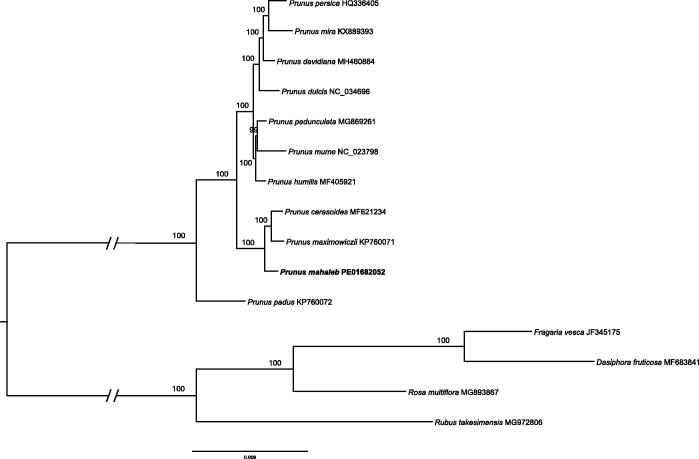
Phylogenetic tree based on plastid genomes using the ML method. Ultrafast bootstrap (UFBoot) values are shown above the nodes, with 1000 bootstrap replicates.

The plastid genomes database consisted of fifteen Rosaceae species, including eleven species of *Prunus* and four species of Rosoideae (outgroup) were used to validate the phylogenetic position of *P. mahaleb*. The sequences were aligned using MAFFT version 7 (Katoh and Standley 2013). ModelFinder (Kalyaanamoorthy et al. 2017) was used for model selection according to the Bayesian information criterion (BIC), and a maximum-likelihood (ML) tree with 1000 bootstrap replicates was inferred by IQ-TREE (Nguyen et al. 2015). *Prunus mahaleb* is located at the base of the subgenus *Cerasus* branch, as the sister group of *P. cerasoides* and *P. maximowiczii* (100% ultrafast bootstrap support) (Hoang et al. 2018) (Figure 1). The complete cp genome of *P. mahaleb* is can be used for phylogenetic analyses, population genomic studies and cp genetic engineering studies of Rosaceae.
